# Cytoplasmic cyclin D1 controls the migration and invasiveness of mantle lymphoma cells

**DOI:** 10.1038/s41598-017-14222-1

**Published:** 2017-10-24

**Authors:** Simon Body, Anna Esteve-Arenys, Hadjer Miloudi, Clara Recasens-Zorzo, Guergana Tchakarska, Alexandra Moros, Sophie Bustany, Anna Vidal-Crespo, Vanina Rodriguez, Régis Lavigne, Emmanuelle Com, Isolda Casanova, Ramón Mangues, Oliver Weigert, Alejandra Sanjuan-Pla, Pablo Menéndez, Bénédicte Marcq, Jean-Michel Picquenot, Patricia Pérez-Galán, Fabrice Jardin, Gaël Roué, Brigitte Sola

**Affiliations:** 1 Normandie Univ, INSERM U1245, UNICAEN, UNIROUEN, Caen, Rouen, France; 2 Division of Haematology and Oncology, IDIBAPS, Barcelona, Spain; 3 Plateforme protéomique - Protim, Biogenouest, Rennes, France; 4 Grup d’Oncogènes i Antitumorals, lnstitut d’Investigacions Biomèdiques Sant Pau and Centro de Investigación Biomédica en Red, Barcelona, Spain; 5 Josep Carreras Leukaemia Research Institute, Department of Biomedicine, School of Medicine, University of Barcelona, Barcelona, Spain; 6 Medical Department III, University Hospital, Ludwig Maximilians University, Munich, Germany; 7 Hematology Service, Hospital Universitario y Politécnico La Fe, Valencia, Spain; 8 Institució Catalana de Recerca i Estudis Avançats, and Centro de Investigación Biomédica en Red de Cáncer, ISCIII, Barcelona, Spain; 9 Service d’anatomie et cytologie pathologiques, Centre Henri Becquerel, Rouen, France; 10 Département d’hématologie clinique, Centre Henri Becquerel, Rouen, France; 11 Laboratory of Experimental Hematology, Department of Hematology, Vall d’Hebron Institute of Oncology, Vall d’Hebron University Hospital, Barcelona, Spain; 12 Present Address: Cytogenetics Laboratory, Research Institute, McGill University Health Centre, Montreal, Canada

## Abstract

Mantle cell lymphoma (MCL) is a hematologic neoplasm characterised by the t(11;14)(q13;q32) translocation leading to aberrant cyclin D1 expression. The cell functions of cyclin D1 depend on its partners and/or subcellular distribution, resulting in different oncogenic properties. We observed the accumulation of cyclin D1 in the cytoplasm of a subset of MCL cell lines and primary cells. In primary cells, this cytoplasmic distribution was correlated with a more frequent blastoid phenotype. We performed immunoprecipitation assays and mass spectrometry on enriched cytosolic fractions from two cell lines. The cyclin D1 interactome was found to include several factors involved in adhesion, migration and invasion. We found that the accumulation of cyclin D1 in the cytoplasm was associated with higher levels of migration and invasiveness. We also showed that MCL cells with high cytoplasmic levels of cyclin D1 engrafted more rapidly into the bone marrow, spleen, and brain in immunodeficient mice. Both migration and invasion processes, both *in vivo* and *in vitro*, were counteracted by the exportin 1 inhibitor KPT-330, which retains cyclin D1 in the nucleus. Our data reveal a role of cytoplasmic cyclin D1 in the control of MCL cell migration and invasion, and as a true operator of MCL pathogenesis.

## Introduction

The *CCND1* gene encoding cyclin D1 is the second most frequently amplified locus in solid cancers^1^. This gene is overexpressed in haematological cancers, due to t(11;14)(q13;q32) translocation, amplification of the *CCND1* gene, deletions or point mutations of the *CCND1* 3′-UTR, and even in the absence of any detectable genetic alteration^2^. Consistent with the well-known role of cyclin D1 in regulating the cell cycle through cyclin-dependent kinase (CDK)4/6 activation, tumour cells with high levels of cyclin D1 have high proliferation rates, linked to a lower nutrient requirement. However, the oncogenic function of cyclin D1 is unlikely to be solely due to an increase in proliferation. Indeed, depending on its subcellular distribution (nuclear, cytoplasmic, at the outer mitochondrial membrane) and its partners (transcription factors, chromatin-modifying enzymes, cytosolic proteins), cyclin D1 can regulate DNA damage response^3,4^, chromosome duplication and stability^5,6^, senescence^7^, mitochondrial function^8,9^ and migration^10–12^, all key biological processes for cancer initiation and maintenance.

In mantle cell lymphoma (MCL), an aggressive form of non-Hodgkin B-cell lymphoma, cyclin D1 is aberrantly expressed due to the t(11;14)(q13;q32) translocation, resulting in the localisation of this protein principally in the nucleus, where it controls tumour cell proliferation^13^. However, we observed a preferential accumulation of cyclin D1 in the cytosolic compartment in a subset of primary tumour samples and cell lines. The accumulation of cyclin D1 in the cytoplasm is associated with the aggressive blastoid variant of MCL. It remains unclear whether cyclin D1 has other oncogenic functions in addition to its role in controlling cell proliferation. We investigated the role of cytosolic cyclin D1 in MCL cells, by performing a proteomic screen for cyclin D1 partners. An analysis of the proteins interacting with cyclin D1 revealed that cyclin D1 bound factors were involved in cell migration, invasion and adhesion. We also found that cytoplasmic cyclin D1 was associated with higher levels of migration and invasion, *in vitro* and *in vivo*.

The migration and invasiveness of MCL cells can be reduced by treatment with KPT-330, a small inhibitor of nuclear export (SINE), which retains cyclin D1 in the nucleus. We suggest that SINEs could potentially be used as single agents or in combination with current drugs, as a promising therapeutic approach for invasive/metastatic MCL.

## Results

### Cyclin D1 accumulates in the cytoplasm in a subset of MCL lines and primary tumours

**Figure 1:**
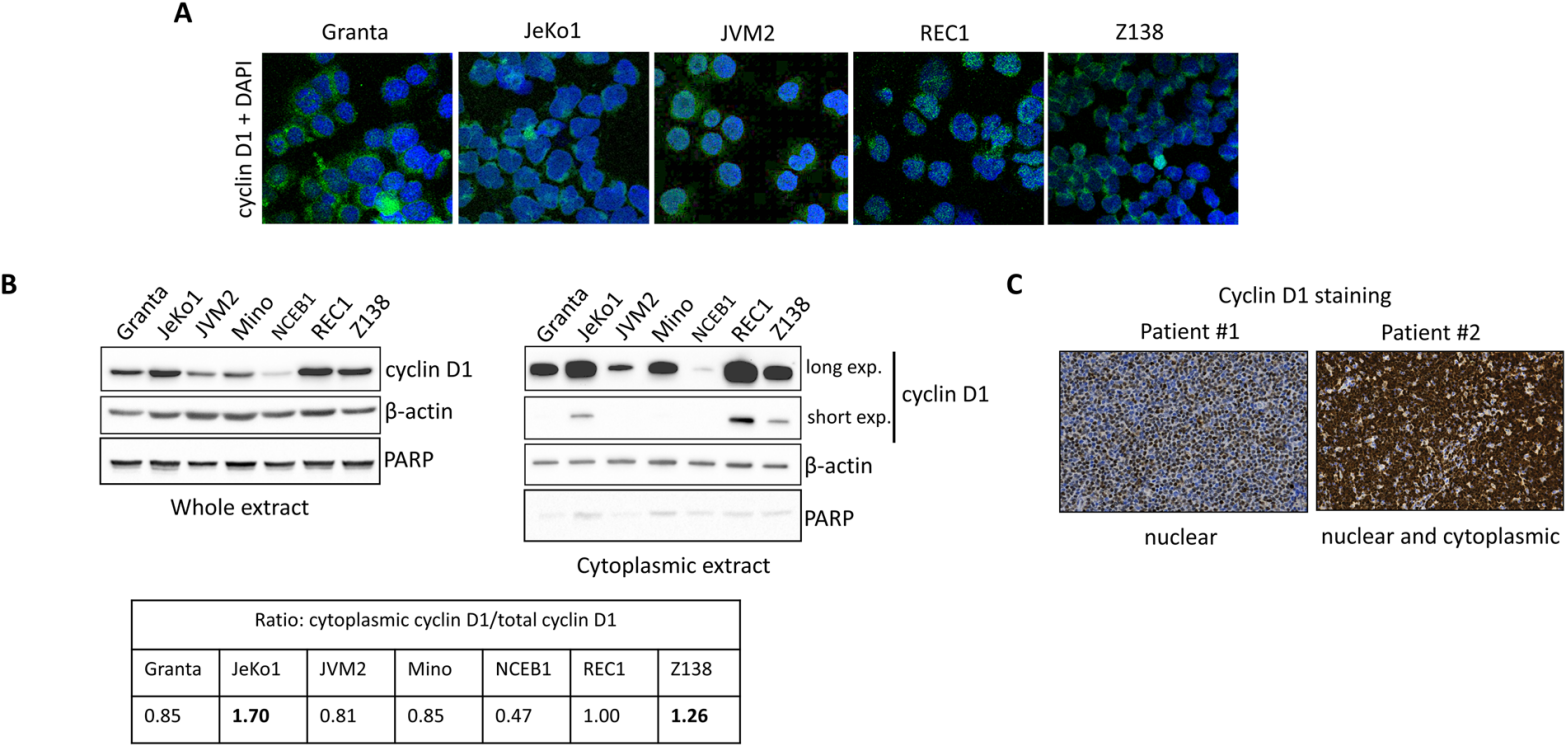
Cyclin D1 may be preferentially distributed in the cytoplasm. (**A**) MCL cells were cytospun on glass slides, stained with an anti-cyclin D1 primary Ab and a goat Alexa Fluor® 488 conjugate anti-mouse IgG as secondary Ab, and counterstained with DAPI. The slides were analysed by confocal microscopy (x 180, magnification). (**B**) Upper panel, cultured MCL cells were harvested. Whole-cell and cytosolic extracts were obtained, subjected to SDS-PAGE and analysed by IB. The same blot was cut into strips and incubated with the indicated Abs. Anti-β-actin Ab was used as a loading control. An anti-PARP Ab was used to verify the purity of cytoplasmic extracts. Lower panel, IB experiments were carried out three times. Total and cytoplasmic cyclin D1 levels were estimated by densitometry (ChemiDoc XRS+, ImageLab software, Bio-Rad). The background of each image was subtracted from the bands of interest, then the density of each protein of interest was normalized against the density of control housekeeping β-actin protein. The ratio of cytoplasmic to total cyclin D1 level was calculated for each cell line for each gel. The mean values are reported in the table. (**C**) Cyclin D1 staining of two biopsy specimens from MCL patients (x20, magnification).

We analysed the subcellular distribution of cyclin D1 in MCL cells by immunofluorescence (IF), which revealed considerable heterogeneity between cell lines. Cyclin D1 was found mostly in the nucleus and partly in the cytoplasm of JVM2, Granta-519 (hereafter referred to as Granta), and REC1 cells, but mostly in the cytoplasmic compartment of JeKo1 and Z138 cells (Fig. 1A). These distributions of cyclin D1 were checked by immunoblotting (IB) purified cytoplasmic/nuclear fractions and total extracts (Fig. 1B, upper panel and Fig. S1A). JeKo1 and Z138 cells had the highest cytoplasmic cyclin D1 contents, whereas this protein was found in both the cytoplasmic and nuclear compartments in REC1 cells. In the other cells, cyclin D1 was mostly present in the nucleus. We estimated the ratio of cytoplasmic to total cyclin D1 by densitometry (Fig. 1B, lower panel). We confirmed the preferential cytoplasmic localisation of cyclin D1 in JeKo1 and Z138 cells, in which this ratio exceeded 1. We then investigated whether such heterogeneity in subcellular distribution was observed in primary cells from MCL patients. IB revealed an accumulation of cyclin D1 in the cytoplasm of primary tumour cells in one (#6414) of three MCL patients tested (Fig. S1B). A retrospective immunohistochemistry (IHC) analysis on a cohort of 42 MCL patients followed at a single institution confirmed that staining for cyclin D1 defined two groups of patients: those with mostly nuclear staining (e.g. patient #1) and those with both nuclear and cytoplasmic staining (e.g. patient #2, Fig. 1C). The predominance of a cytoplasmic pool cyclin D1 is, thus, not rare in MCL.

### Cytoplasmic cyclin D1 is a marker of MCL aggressiveness

We investigated the clinical relevance of the subcellular localisation of cyclin D1, by analysing clinical and histological features at the time of initial diagnosis, in the same cohort of patients with MCL. Overall, 6/42 (14%) cases presented both nuclear and cytoplasmic immunostaining for cyclin D1, whereas 36/42 (86%) displayed immunostaining limited to the nucleus. Cyclin D1 expression was never found only in the cytoplasm. Patients with cytoplasmic immunostaining for cyclin D1 were significantly more likely to have been diagnosed with the blastoid MCL variant^14^ (33% *vs*. 3%, *p* = 0.04). All these patients had a Ki-67 index of more than 10%, suggesting a more proliferative disease (Table 1). For example, patient #2 (Fig. 1C) was diagnosed with a blastoid variant on the basis of morphological criteria and had a Ki-67 value of 66%. By contrast, the two groups did not differ significantly for the principal clinical features considered, such as splenomegaly, leukocytosis and bone marrow (BM) involvement (Table 1). The limited number of cases and the diverse treatments administered to the patients of this cohort precluded an analysis of overall and disease-free survival.

**Table 1:** Correlation between clinical/histological features and cyclin D1 localization for MCL patients.

MCL patients	Cyclin D1 immunostaining		*p*
	Nucleus	Nucleus/cytoplasm	
	n = 36	n = 6	
**Clinical features**			
Median age (range)	68 (40–85)	54 (33–72)	0.101
Male (%)	18/35 (51%)	5/6 (83%)	0.20
Median MIPI^a^ (range)	7.2 (4.8–10.7)	8.6 (5.3–10)	0.92
BM involvement	23/33 (69%)	3/6 (50%)	0.38
Stage IV (%)	28/33 (84%)	4/6 (66%)	0.29
Leukocytosis > 10 Giga/L (%)	11/33 (33%)	3/6 (50%)	1
Splenomegaly (%)	11/33 (33%)	2/6 (33%)	1
**Histological features**			
Ki-67 > 10%	22/36 (61%)	**6/6 (100%)**	0.15
Blastoid variant	1/36 (3%)	2/6 (33%)	**0.048**

^a^MIPI, mantle cell lymphoma international prognostic index. *p* was calculated in Fisher’s exact test. *p* < 0.05 was considered significant.

### A proteomic-based interactome analysis reveals an extensive network of cyclin D1-interacting proteins

We investigated the main molecular functions of cytosolic cyclin D1 in MCL, by trying to identify factors interacting with cyclin D1 in JeKo1 cells. The partners of cyclin D1 may be common to different cyclin D1-expressing tumour cells^4^. We therefore included, for comparison, the U266 multiple myeloma (MM) cell line, in which *CCND1* is activated by the insertion of an enhancer element^15^. In these cells, cyclin D1 is overexpressed and present in both in the nucleus and the cytoplasm^9^. Cytosol-enriched extracts were purified from both cell lines, subjected to immunoprecipitation with an anti-cyclin D1 antibody (Ab), and the cyclin D1-containing complexes obtained were subjected to mass spectrometry. Proteomic data revealed a large number of putative cyclin D1-interacting factors in each cell line (Tables S1 and S2) in addition to cyclin D1 itself. For validation of our protocol, we compared these two sets of proteins with available datasets for the Granta MCL cell line^4^. We identified 66 proteins strictly identical in the three cell lines, and 17 belonging to the same family. These proteins were classified on the basis of their cellular functions, with the Database for Annotation, Visualization and Integrated Discovery (DAVID) bioinformatics resources v6.7 (ref.^16^). In addition to well-known cell cycle regulators (CDKs and CDKNs), cyclin D1 was found to interact with proteins involved in metabolism, transcriptional regulation, DNA repair, replication, protein folding, cell structure and organisation (Table S3). Some previously characterised partners of cyclin D1, such as PCNA, RAD51, HDAC and HSPs^17,18^, were also detected in our dataset, validating our technical procedure.

### The set of cytosolic partners of cyclin D1 is enriched in structural proteins

**Figure 2:**
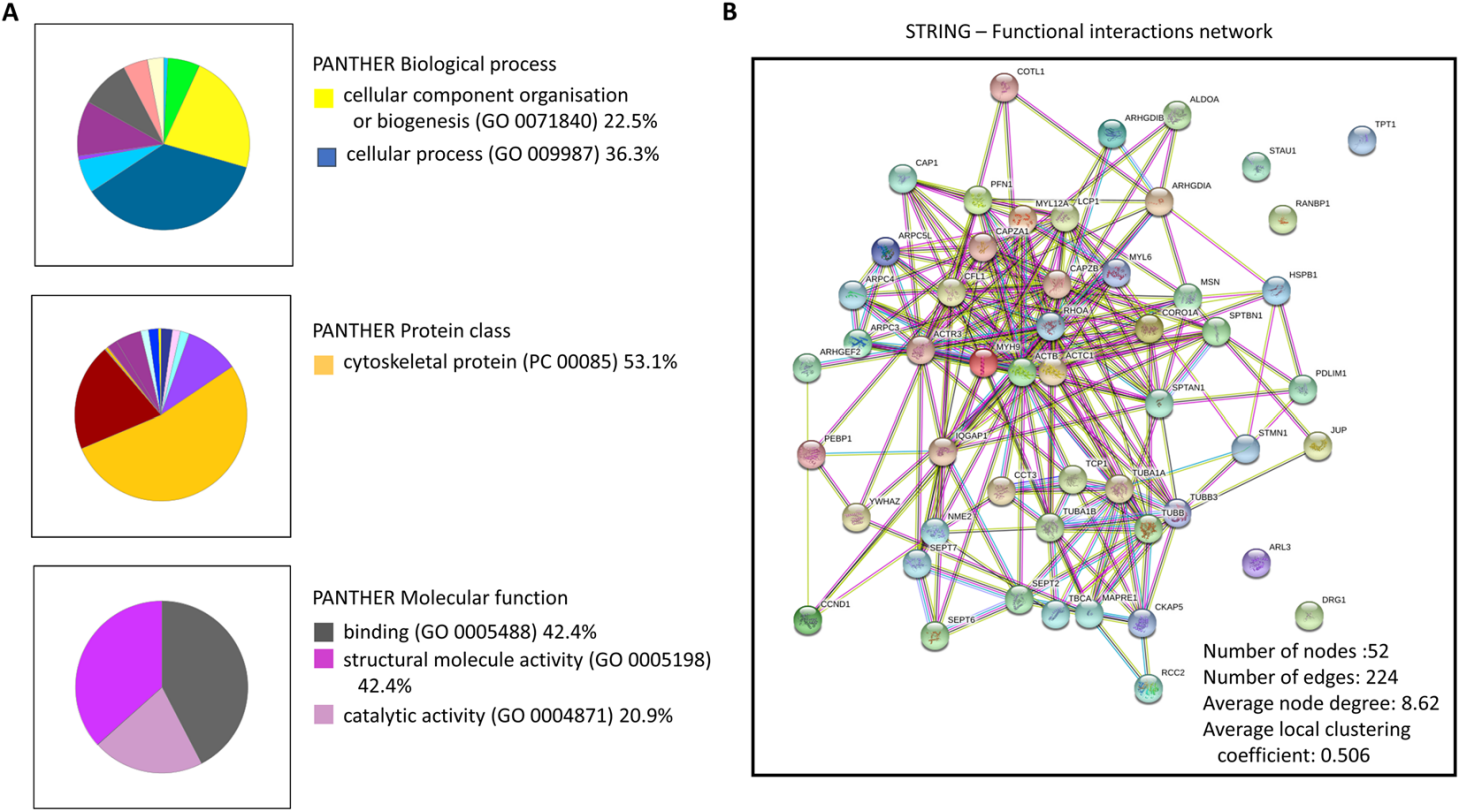
Cytoskeleton-associated proteins interact with cyclin D1 in JeKo1 cells. The 200 proteins with the highest peptide coverage (Table S4) were analysed by DAVID Bioinformatics Resources v6.7 (ref.^16^). Relevant proteins were uploaded using their UniProt ID and organised into functional groups by the tool, according to GO terms. Among them, 51 were cytoskeleton-associated proteins (enrichment score: 13.16, *p*-value: 4.7 × 10^−14^). (**A**) The same 200 proteins were next clustered with the PANTHER™ classification system v12.0 according to their associated biological processes, molecular functions and protein classes (www.pantherdb.org, ref.^19^). Results are presented as pie charts, with the percentage of proteins for some of the more relevant fractions. (**B**) The network designed with STRING (v10.5, www.string-db.org, ref.^20^) summarises associations between the 51 proteins previously characterised and cyclin D1. This analysis predicted functional enrichment in actin-filament-based processes (pathway ID: GO 0030029), with a false discovery rate of 7.69 × 10^−11^.

For identification of the most relevant cyclin D1-interacting factors in JeKo1 cells, we performed functional clustering with DAVID tools^16^ on the 200 proteins associated with cyclin D1 with the highest peptide coverage (Table S4). Interestingly, 51 of these proteins were cytoskeleton-associated proteins (enrichment score: 13.16, *p*-value: 4.7 × 10^−14^). We then classified the same 200 proteins with the PANTHER™ system (v12.0, ref.^19^) on the basis of biological process, molecular function and protein class. The results are presented as pie charts, with the percentage of proteins within some relevant fractions. These results confirmed the DAVID findings (Fig. 2A). The network obtained with STRING (v10.5, ref.^20^) summarised interactions between the previously characterised 51 proteins and cyclin D1. This analysis predicted a functional enrichment for the actin-filament-based process (pathway ID: gene ontology (GO) 0030029), with a false discovery rate of 7.69 × 10^−11^ (Fig. 2B). Immunoprecipitation (IP) experiments confirmed the binding of cyclin D1 to α-tubulin and β-actin proteins in JeKo1 cells (Fig. S1C), suggesting a potential functional role of cyclin D1 in the control of actin fibre organisation and microtubule dynamics. B cells transduced to overexpress a TAT-cyclin D1 fusion protein^9^, displayed ultrastructural changes, such as a loss of protrusions (blebs) and a more regular cell surface (Fig. S1D), suggesting a link between cyclin D1 and cellular shape remodelling, which is crucial for the migratory/invasiveness phenotype.

### Cytoplasmic cyclin D1 controls MCL migration and invasion, but not adhesion

Based on the potential role of cyclin D1 in regulating cellular shape through binding to structural proteins, we investigated the link between the processes of adhesion, migration and invasion, and cytoplasmic levels of cyclin D1.

The crosstalk between MCL cells and the stromal microenvironment in the BM and secondary lymphoid tissues plays an important role in the biology of the disease and is known to contribute to drug resistance^21^. In particular, MCL cells have high levels of adhesion molecules enabling them to interact with fibronectin and stromal cells^22^. We therefore assessed the adhesion properties of MCL cells with fibronectin and HS-5 mesenchymal stromal cells. We found no correlation between cytosolic cyclin D1 level and the number of cells adhering to either fibronectin or HS-5 cells (Fig. S2A), ruling out a role for cytoplasmic cyclin D1 in MCL cell adhesion.

**Figure 3:**
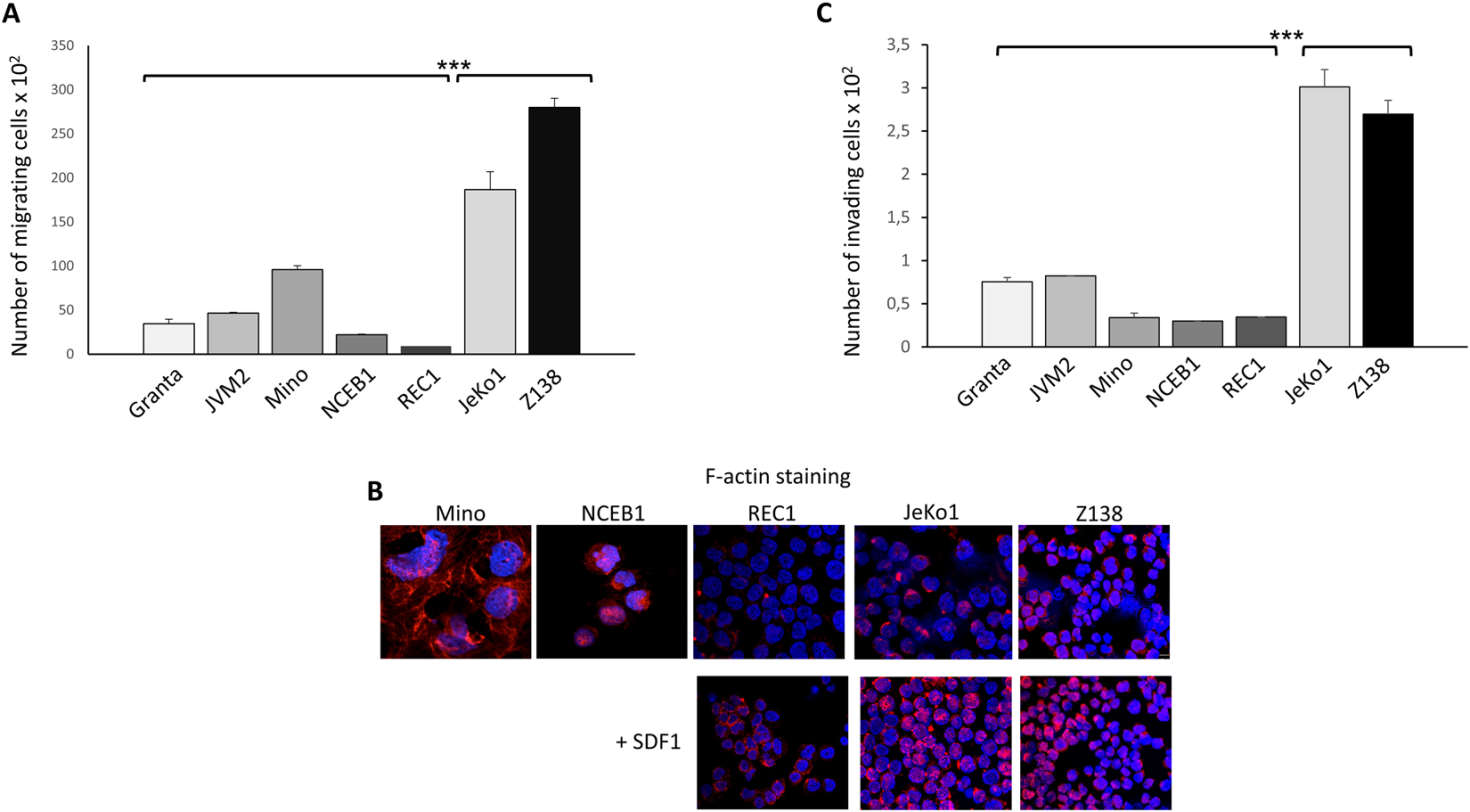
Cytoplasmic cyclin D1 controls the invasion and migration of MCL cells *in vitro*. (**A**) MCL cells were used to seed the top chamber of Transwell inserts. The inserts were transferred into wells containing medium supplemented with SDF1 and the cells were allowed to migrate for 4 h. The cells present in the bottom well were then counted. Three independent inserts were used per experiment and each experiment was performed three times. The mean numbers of migrating cells ± s.d. are plotted on the graph. ***p < 0.001 in Student’s *t*-test. (**B**) MCL cells were left untreated (controls) or treated with SDF1 (200 ng/ml for 4 h), then cytospun, fixed and permeabilized. F-actin was visualised with rhodamine-stained phalloidin by confocal microscopy (Fluoview FV 1000 confocal microscope and Fluoview Viewer software, Olympus). Nuclei were counterstained with DAPI (x180, magnification). (**C**) Cultured MCL cells were used to seed the ECM-coated 5 μm-pore membranes of Transwell inserts, in the top chamber, containing serum-free medium. The inserts were then placed in wells containing SDF1 as a chemoattractant and incubated for 24 h. The cells invading the lower chamber were counted by flow cytometry. The means ± s.d. of three independent experiments with triplicate samples are indicated on the graph. ***p < 0.001 in Student’s *t*-test.

We assessed the ability of MCL cell lines to migrate over a gradient of stromal cell-derived factor 1 (SDF1), the relevant chemokine for MCL cells in chemotaxis assays^23^. JeKo1 and Z138 cells had a greater migratory capacity than MCL lines in which cyclin D1 was located in the nucleus, and REC1 cells did not migrate (Fig. 3A). This greater migratory capacity was associated with greater filamentous (F-) actin polymerisation and a broader F-actin distribution, as shown by rhodamine-phalloidin staining before and after stimulation with SDF1 (Fig. 3B). In addition to playing a role in cell migration^10–12,24^, cyclin D1 has been associated with tumour invasiveness and metastasis^25^. Consistent with these findings, we observed that MCL cell lines with cytosolic cyclin D1 were more invasive in extracellular matrix (ECM)-coated invasion chambers and on an SDF1 gradient (Fig. 3C). These findings are supported by the results of a recent study showing that a cytoplasmic and membrane-associated form of cyclin D1 controls the invasiveness of tumour cells^26^.

### MCL cell lines have different engraftment kinetics in xenograft models

**Figure 4:**
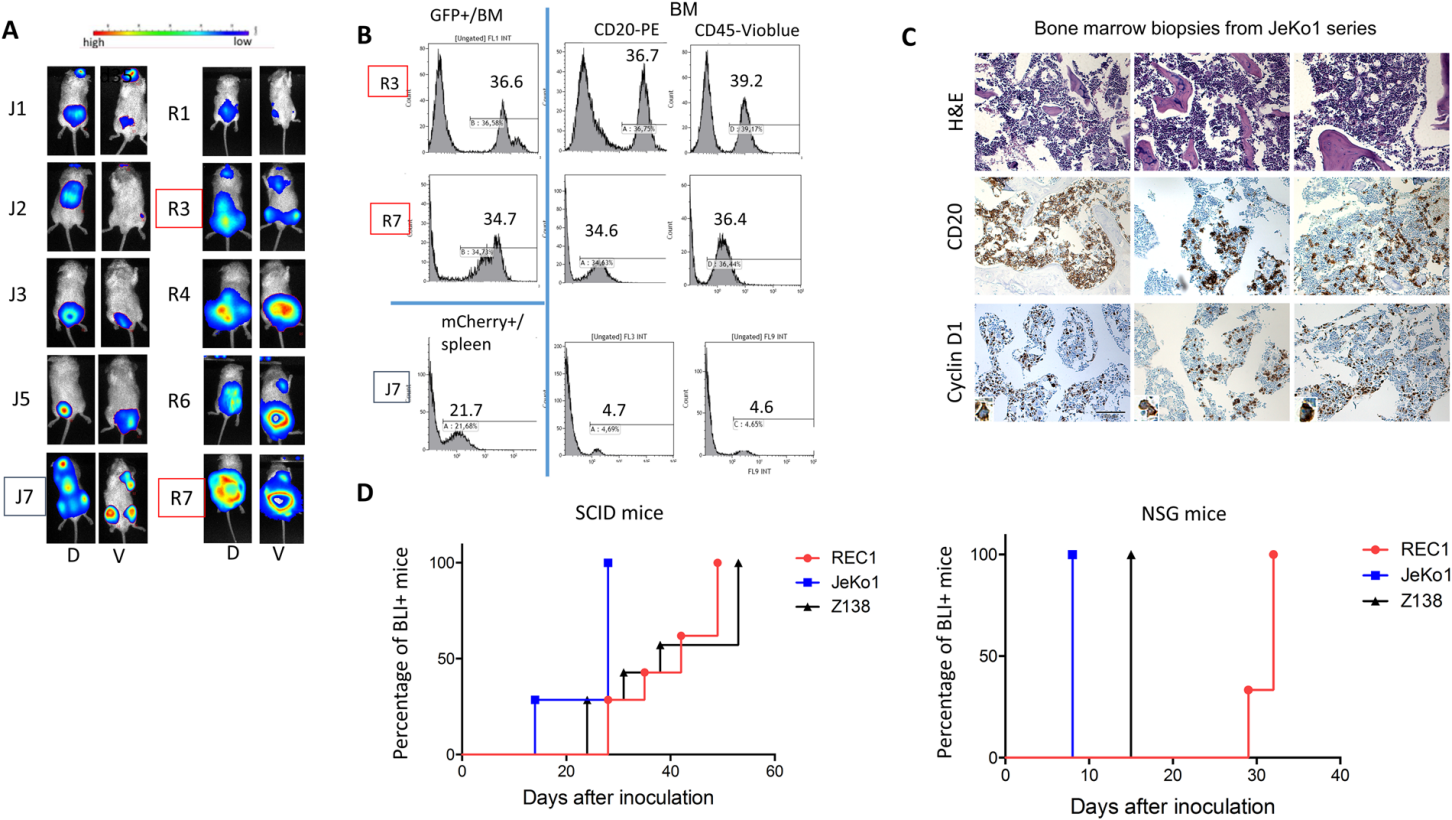
MCL cells display variable engraftment properties. (**A**) We injected 10^7^ MCL cells into the caudal veins of six-week-old SCID mice, and BLI analyses were performed, starting one week after cell injection. Mice receiving JeKo1-mCherry-Luc (J) or REC1-GFP-Luc (R) cells underwent imaging on the dorsal (D) and ventral (V) sides at successive time points. Images obtained 35 days after cell injection (at the time of death) are presented. (**B**) Cells were isolated from the BM and spleen and fluorescence signals were recorded by flow cytometry. The percentages of GFP^+^ or mCherry^+^ fluorescent cells on flow cytometry profiles are shown for R3, R7 and J7 mice. Isolated cells were also stained with anti-CD20-PE and anti-CD45-Vioblue Abs and analysed by flow cytometry. The numbers of CD20^+^ or CD45^+^ cells in the bone marrow of R3, R7 and J7 mice are indicated on the figure. (**C**) Haematoxylin/eosin staining (H&E) and IHC analyses of BM biopsy specimens from JeKo1-inoculated mice showing the presence of CD20^+^ and cyclin D1^+^ human tumour cells (x200, magnification). (**D**) The percentages of SCID mice (left panel) and NSG mice (right panel) displaying luciferase activity as a function of time after injection are presented on Kaplan-Meyer curves drawn with Prism v6.0 software (GraphPad).

We then investigated the possible association of a cytoplasmic cyclin D1 distribution with greater invasiveness in MCL cells. We engineered three cell lines from JeKo1, Z138 and REC1 cells for expressing the green fluorescence protein (GFP) or mCherry and the luciferase reporter gene (see details in the Supplementary Information). The three cell lines (JeKo1-mCherry-Luc, Z138-GFP-Luc and REC1-GFP-Luc) were injected into the tail vein of either SCID or NOD/SCID/IL2Rγ^null^ (NSG) immunodeficient mice. Tumour growth was monitored weekly by bioluminescence imaging (BLI) after the i.p. injection of D-luciferin. Indeed, areas of luciferase activity are the sign of tumour growth. Based on sequential BLI measurements, tumours grew exponentially overtime, for the whole mouse as well as for individual luminescent foci (data not shown). The three cell lines invaded their pathophysiological niches, including the spleen and BM, and non-haematopoietic tissues, such as the brain and gut, with 100% penetrance (as shown in Fig. 4A and Table S5), thereby faithfully reproducing the features of MCL disease. Organ specimens showing luciferase activity contained tumour cells as assessed by flow cytometry analysis of GFP+/mCherry+ and CD20+/CD45+ cells (Fig. 4B). IHC on representative BM biopsy specimen confirmed the presence of cyclin D1+/CD20+ tumour cells. Representative tumour sections from the Jeko1 series showed an intense cytoplasmic cyclin D1 staining (Fig. 4C). Interestingly, marked differences were observed between the three cell lines in terms of lymphoid tissues-homing kinetics. Indeed, JeKo1 and Z138 cells had a higher engraftment capacity in NSG mice, and, to a lesser extent, SCID mice, than REC1 cells, and this engraftment also occurred earlier (Fig. 4D, Table S5). We thus conclude that the accumulation of cyclin D1 in the cytoplasmic compartment is associated with a higher invasion capacity *in vivo*.

### The nuclear retention of cyclin D1 alters the invasiveness/migratory properties of MCL cells *in vitro*

**Figure 5:**
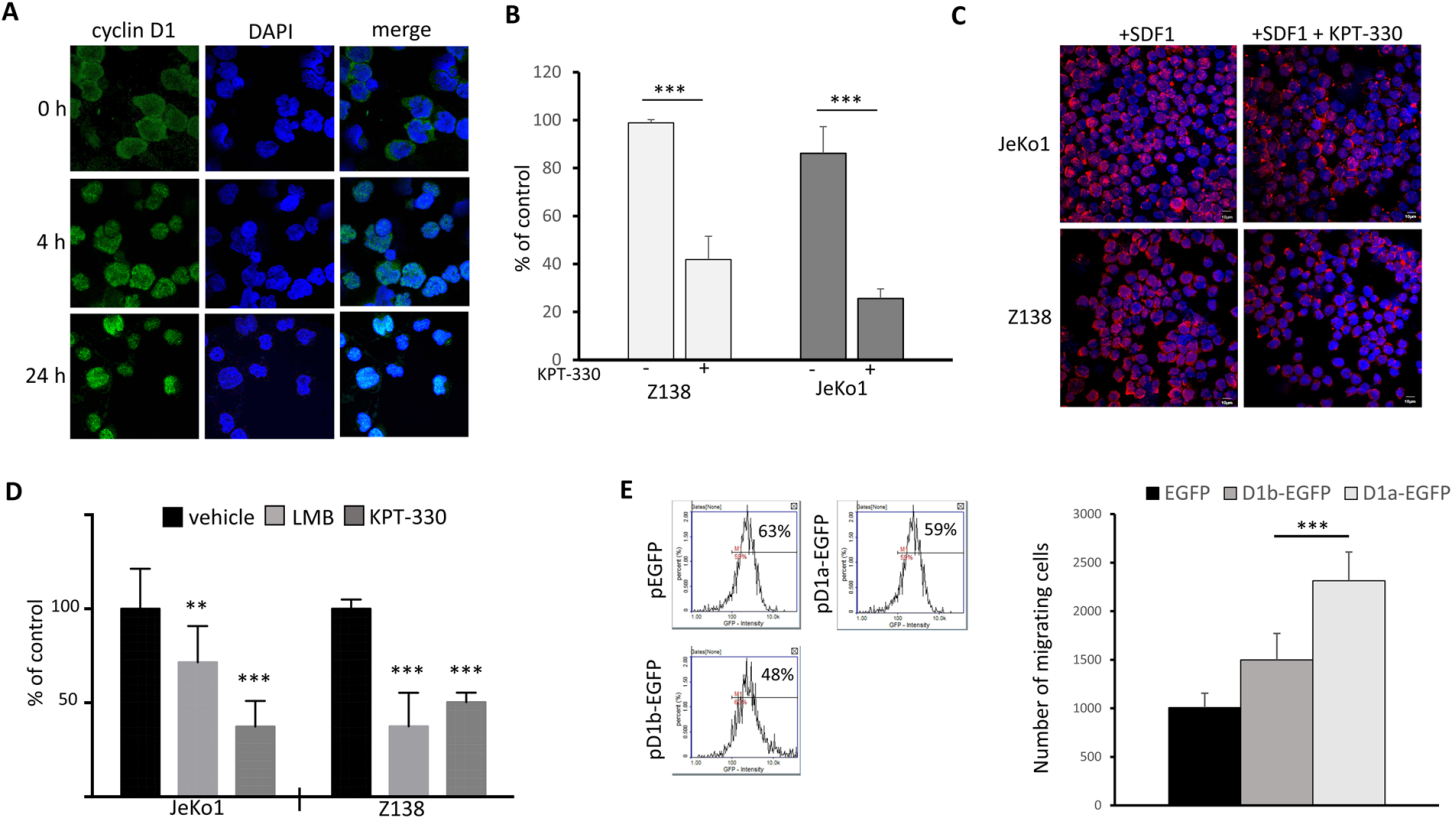
The inhibition of cyclin D1 export impairs both the migration and invasion of MCL cells *in vitro*. (**A**) JeKo1 cells were treated with 300 nM KPT-330 and analysed by IF after 4 h or 24 h, to determine the distribution of cyclin D1. We used an anti-cyclin D1 as primary Ab and a goat Alexa Fluor® 488 conjugated anti-mouse IgG secondary Ab. Slides were counterstained with DAPI and analysed by confocal microscopy (x180, magnification). (**B**) JeKo1 and Z138 cells cells were treated with vehicle or KPT-330 (300 nM for 4 h) and then used to seed the upper chamber of Transwell inserts. The inserts were transferred to medium containing SDF1 and the cells were allowed to migrate for 4 h. The cells present in the bottom well were then counted. The data were normalised as a percentage of the control value. Three independent inserts were used per experiment and the experiment was performed three times. The mean numbers of migrating cells ± s.d. are plotted on the graph. ***p < 0.001 in Student’s *t*-tests. (**C**) F-actin staining with rhodamine-phalloidin was performed on JeKo1 and Z138 cells stimulated with 200 ng/ml SDF1 for 4 h alone or together with 300 nM KPT-330. Cells were counterstained with DAPI before examination (x 180, magnification). (**D**) JeKo1 and Z138 cells were treated with 5 ng/ml LMB, 300 nM KPT-330, or vehicle as a control for 4 h and then assayed for invasion, as described above. The data were normalized as a percentage of the control value. The experiment was carried out three times (LMB), or twice (KPT-330), with triplicate samples. **p < 0.01; ***p < 0.001 in Student’s *t*-tests. (**E**) JVM2 cells were transfected with either pEGFP, pD1a-EGFP or pD1b-EGFP expression plasmids using the Nucleofector II device (solution V, program A-023). After a 48 h-recovery period, the transfection efficiencies were estimated using the NucleoCounter NC-3000. Transfected cells were assayed for migration as described above. The experiment was performed twice with quadruplicate samples. The means ± s.d. are indicated on the graph, ***p < 0.001 in *t*-tests.

Cyclin D1 is a cargo of XPO1, a major nuclear export protein^27^. We found that XPO1 was strongly expressed in both in the nuclear and cytosolic compartments of MCL cells, as expected from its function as a nuclear exportin (Fig. S2B). We treated JeKo1 cells with the SINE, KPT-330 and followed the redistribution of cyclin D1 over time. Cyclin D1 was strictly nuclear by 4 h after SINE treatment and remained nuclear 24 h later (Fig. 5A). The migratory capacities of JeKo1 and Z138 cells were analysed after a KPT-330-treatment. For both cell lines, significantly fewer cells migrated when cyclin D1 was retained into the nuclear compartment (Fig. 5B). Moreover, the blockade of cyclin D1 export from the nucleus led to a clear inhibition of F-actin polymerisation and changes in F-actin distribution (Fig. 5C). Similarly, both KPT-330 and leptomycin B (LMB), another SINE, decreased the invasiveness of these two cell lines (Fig. 5D). To further confirm these data, the JVM2 cell line that has low levels of cyclin D1 expression (Fig. 1B) was chosen as the recipient for transient transfections. Three kinds of plasmids were transfected by electroporation: p-EGFP encoding tGFP as a control, pD1a-EGFP encoding the canonical long form of cyclin D1 and pD1b-EGFP encoding a short isoform of cyclin D1. This short isoform lacks the Thr286 residue that is necessary for nuclear export by XPO1 and degradation by the ubiquitin/proteasome system^2^. In turn, cyclin D1b accumulates in the nucleus. We assessed the capacity of transfected cells to migrate in the chemotaxis assay. As shown Fig. 5E, cyclin D1 expression increased the migration capacity of cells. However, this increased capacity is statistically higher for the long cyclin D1 isoform than the short nuclear isoform. Cytoplasmic cyclin D1 thus seems to be required to promote chemotaxis and MCL cell invasion *in vitro*.

### The nuclear relocalisation of cyclin D1 alters the invasiveness/migratory properties of MCL cells *in vivo*

**Figure 6:**
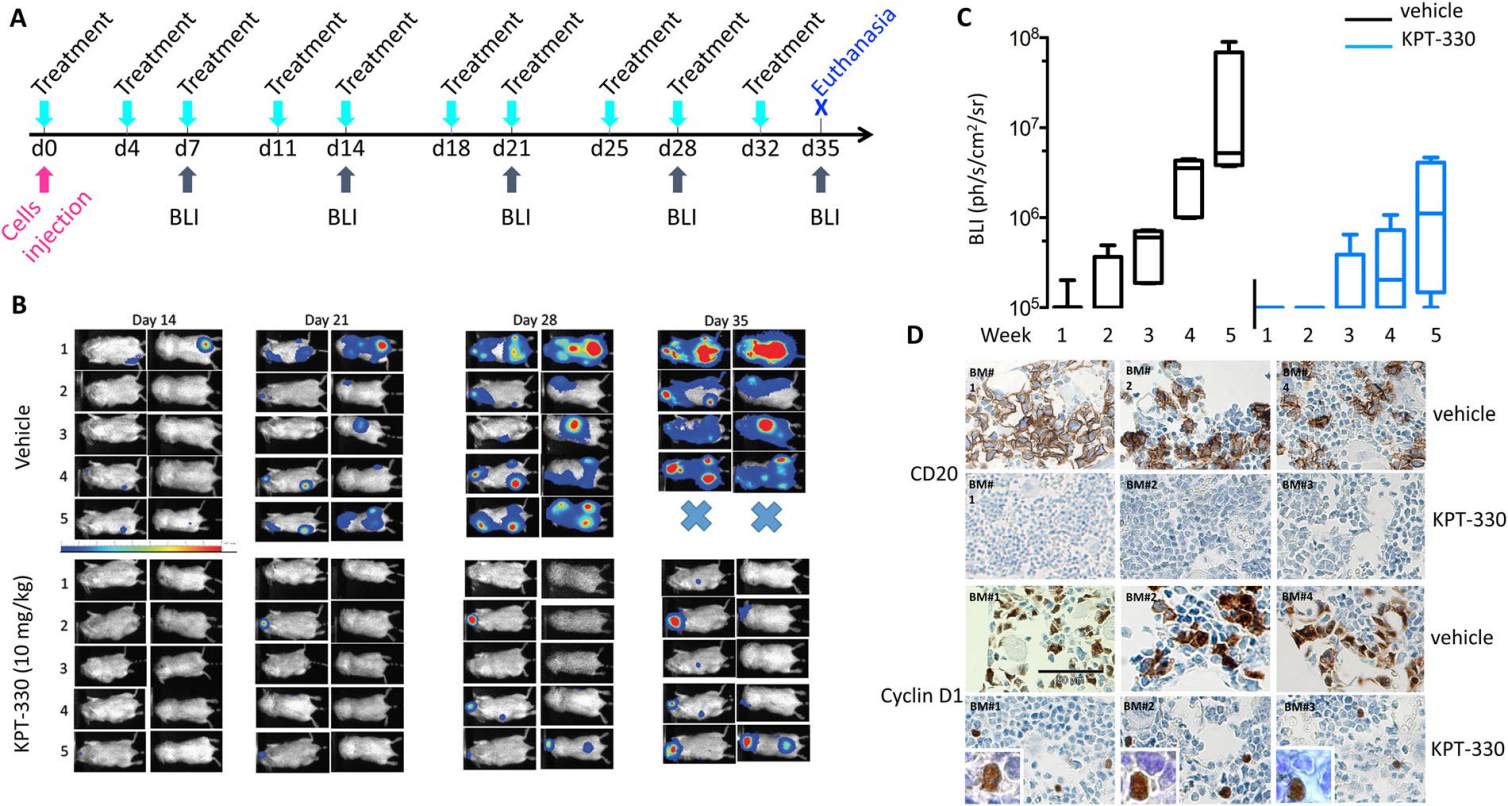
The inhibition of cyclin D1 export impairs MCL engraftment *in vivo*. (**A**) We injected 10^7^ mCherry-Luc-JeKo1 cells i.v. into six-week-old male SCID mice (*n* = 10). Mice were randomised to two groups (*n* = 5). One group received KPT-330 (10 mg/kg) orally twice weekly, whereas the other group received vehicle i.p. Both treatments began on the day of cell injection and BLI analyses began one week later. Mice were imaged on the dorsal and ventral sides once weekly after the i.p injection of D-luciferin. Images of individual mice (1–5) from both groups (KPT-330 and vehicle) are presented. Mice were killed after the last BLI images were obtained (day 35). (**B**) The graph shows the Tukey boxplots for individual BLI data as a function of time after cell injection for the two groups. (**C**) At the end of the experiment, the blood, spleen, lower limbs and brain of each mouse were collected and tumour cells were analysed by IHC. Staining for cyclin D1 and CD20 on BM biopsy specimens of one mouse per group are shown. Enlarged boxes: representative cyclin D1 nuclear staining (x800, magnification).

For validation of the *in vitro* data, SCID mice (*n* = 5 per group) received intravenous injections of JeKo1-mCherry-Luc cells. They were then treated twice weekly with KPT-330 or vehicle and analysed by BLI once weekly (Fig. 6A). Tumour cells grew exponentially over time in both groups, but with a very different kinetics (Fig. 6B and C). Indeed, BLI results were positive in three of the five mice in the control group as soon as day 14 after the injection of the cells, and all mice displayed luciferase activity at day 21, with a pattern consistent with BM infiltration (femur, tibia, rachis areas). By contrast, on day 21, luciferase activity was detected in only two of the five mice in the KPT-330-treated group, and the BLI signal was restricted to extramedullary organs, such as the brain and abdominal regions. Flow cytometry confirmed that spleen and BM biopsy specimens from animals treated with vehicle were enriched in CD20^+^/CD45^+^ tumour cells, whereas tumour cells were detected in the brains of only two mice and in the spleen of only one mouse treated with KPT-330 (Table 2). Accordingly, tumour cell infiltration into the BM was almost completely prevented in KPT-330-treated mice, as shown by IHC labelling for cyclin D1 and CD20 cells in representative biopsy specimens from both groups (Fig. 6D). Interestingly, in the few surviving CD20^+^ tumour cells, cyclin D1 accumulated in the nucleus (compare Fig. 6D and Fig. 3C). Thus, the nuclear retention of cyclin D1 delayed the onset of MCL cells engraftment, impaired tumour growth and altered tumour cells homing towards the lymphoid compartments. These *in vivo* data confirm the crucial role of the subcellular distribution of cyclin D1 In tumour cell engraftment and invasion.

**Table 2:** Percentage of tumour cells in various organs from control (C1-5) and KPT-330 treated (K1-5) SCID mice.

Mouse	Organ	% of CD20^+^ cells	% of CD45^+^ cells
**C1**	Brain	<1	<1
	BM	33	34
	Spleen	61	64
	Blood	3	nd^a^
**C2**	Brain	<1	<1
	BM	22	21
	Spleen	62	65
	Blood	11	nd
**C3**	Brain	2	2
	BM	53	60
	Spleen	78	79
	Blood	5	nd
**C4**	Brain	2	1
	BM	62	63
	Spleen	78	79
	Blood	4	nd
**C5**	Brain	7	4
	BM	11	10
	Spleen	nd	nd
	Blood	<1	1.0
**K1**	Brain	0	0
	BM	<1	<1
	Spleen	3	<1
	Blood	<1	<1
**K2**	Brain	1	1
	BM	<1	<1
	Spleen	<1	<1
	Blood	<1	<1
**K3**	Brain	2	3
	BM	<1	<1
	Spleen	<1	<1
	Blood	<1	<1
**K4**	Brain	<1	<1
	BM	<1	<1
	Spleen	<1	<1
	Blood	<1	<1
**K5**	Brain	5	6
	BM	<1	<1
	Spleen	<1	<1
	Blood	<1	<1

JeKo1-mCherry-Luc cells were injected into the caudal vein of 10 SCID mice, which were then randomised into two groups: a control group (C series) and a group receiving KPT-330 (K series). K1-5 mice were treated orally, twice weekly, with 1 mg/kg KPT-330. The overall distribution of MCL tumor cells was determined by BLI during the time course of the experiment and analysed by flow cytometry at the time of mouse death (day 35 after cells injection for all mice except C5, which was killed on day 28). Cells were isolated from the organs indicated and stained with CD20-PE or CD45-VioBlue Abs. The percentage of stained cells was deduced from the cytometry profiles analyzed with the Kaluza software v1.5 A. ^a^nd, not determined.

## Discussion

The *CCND1* gene is the second most frequently amplified locus in the human genome^1^ and its product, the cyclin D1 protein, is overexproduced due to various genetic alterations, including translocations, mutations, oncogenic activation, and the deletion of miRNA-binding sites^2^. In association with CDK4/6, cyclin D1 controls the cell cycle and proliferation. Cyclin D1 is an oncogenic driver for several solid tumours and haematological malignancies, but has also non-canonical oncogenic properties, as demonstrated by two previous proteomic screens^4,28^. We carried out a proteomic screen in a cyclin D1-expressing cell model, to characterise the partners of cyclin D1 responsible for these non-canonical oncogenic features. A comparison of cyclin D1 interactomes in MCL cells (JeKo1 and Granta) and in MM cells (U266) revealed that cyclin D1 bound to factors involved in DNA repair (such as PCNA and RAD51), the heat shock response (HSPs, DNAJs), transcriptional regulation (HDACs), and to structural proteins. Most of these cyclin D1 partners in B-cell lymphoma and myeloma cells, are also present in solid tumours (breast cancer, squamous cell carcinoma and colorectal cancer)^4^, further validating our technical protocol. Our data confirm that non-canonical, CDK-dependent and -independent cyclin D1 functions are common to many cancer cells.

More importantly, our analysis of the most frequently identified partners of cyclin D1 in JeKo1 cells revealed a complex network of cytoskeleton-associated proteins. Using *in vitro* and *in vivo* devices, we identified an unexpected function of cyclin D1 in cell remodelling and in turn, migration and invasiveness. This finding is consistent with those of a recent study showing that a cytoplasmic form of cyclin D1 artificially attached to the cell membrane controls invasiveness^29^. Cyclin D1/CDK4 complexes interact and phosphorylate the scaffold protein filamin A, a member of the actin-binding protein family, thereby controlling the invasion potential of breast cancer cells^30^. In prostate cancer cells, cytoplasmic cyclin D1/CDK4 complexes phosphorylate paxillin, a structural component of focal adhesions^29^. Consistent with these findings, we found that the treatment of MCL cells with high levels of cytoplasmic cyclin D1 with the CDK4 inhibitor palbociclib reversed the invasive phenotype (Fig. S2C), suggesting that the kinase activity associated with cyclin D1 is required for MCL cell invasion. However, we found no evidences for the phosphorylation of paxillin, filamin A or cofilin, another actin-associated protein for which phosphorylation by cyclin D1/CDK4 complexes is involved in cell motility^31^ (data not shown). The downstream targets of cytoplasmic cyclin D1/CDK4 dimers regulating migration/invasion remains unknown, but our proteomic data identify the characterized cytoskeleton-associated proteins as possible effectors.

KPT-185 and KPT-286, two XPO1 inhibitors, block the proliferation and induce the apoptosis of MCL cell lines and primary cells *in vitro* ^32,33^. *In vivo*, the oral administration of KPT-276 also suppresses the growth of subcutaneous xenografts tumours^32^. We describe here the effect of KPT-330 in a mouse model reproducing the pathogenic features of human MCL. We found that KPT-330 impaired MCL cells engraftment and migration to the pathophysiologic niches of these cells. Consistent with our data, KPT-330 has been shown to inhibit SDF1-mediated signalling, preventing the migration of CLL cells^34^, and to restrict tumour spreading and the metastasis of prostate cancer cells by decreasing the secretion of metalloproteinases and urokinase^35^.

In KPT-330-treated mice, in addition to the lower engraftment potential of MCL cells, we found that the brain replaced the haematopoietic organs as the most frequently infiltrated organ. We interpret this finding as indicating that alterations occur to the factors required for MCL homing, such as chemokines, chemokine receptors, integrins, and sphingosine 1 phosphate receptors, after the relocalisation of cyclin D1 (and possibly other XPO1 cargos) to the nucleus. Thus, tumour cells avoid haematopoietic organs because they cannot interact with their protective microenvironment. KPT-330 effects on the entry, retention and egress of MCL cells may be indirect and mediated by proteins such as cyclin D1, controlling motility and invasiveness.

Cyclin D1 has a heterogeneous subcellular distribution in MCL cell lines and primary tumour cells, but its presence in the cytoplasm does not mean that it is excluded from the nucleus. The nuclear functions of cyclin D1 (essentially, regulation of the cell cycle and transcription) are therefore maintained in cells with a high cytoplasmic cyclin D1 content. The nuclear relocalisation of cyclin D1 associated with CDK4 has been recognised as a mechanism for controlling proliferation in solid tumours^24,25,30^. Our data, therefore, strongly suggest that tumour cells proliferation may be controlled by nuclear cyclin D1, whereas tumour cells invasiveness may be controlled by the cytoplasmic fraction of the protein. Consistent with this conclusion, Fusté and colleagues reported that the attachment of cyclin D1 at the cytoplasmic membrane of tumour cells had no effect on proliferation but increased tumour invasion and metastasis^29^.

Further studies are required, but our finding of different oncogenic properties associated with different subcellular distributions of cyclin D1 at diagnosis in MCL patients could up new possibilities for personalised treatment. In particular, the combination of KPT-330 or next-generation SINEs with currently available proteasome inhibitors, such as bortezomib or carfilzomib, might decrease the aggressiveness of MCL tumour cells, limiting their spread. The maintenance of malignant cells outside of their niches would leave these cells more accessible to chemotherapy.

## Methods

### Antibodies

Antibodies (Abs) against cyclin D1 (sc-718), β-actin (sc-4778), PARP (poly(ADP-ribose) polymerase, sc-8007) and exportin 1 (XPO1, sc-5595) were purchased from Santa Cruz Biotechnology. An Ab against α-tubulin (T6199) was purchased from Sigma-Aldrich, and an anti-glyceraldehyde-3-phosphate dehydrogenase Ab (GAPDH, clone 6C5) was obtained from Life Technologies. Abs against hexokinase 2 (#2106), and binding immunoglobulin protein (BiP, #3183) were obtained from Cell Signaling Technologies. ImmunoPure peroxidase-conjugated goat anti-rabbit and anti-mouse IgG (H + L) secondary Abs were purchased from Pierce Protein Research Products.

### MCL patients and analysis of cyclin D1 localization

We assessed the clinical relevance of the subcellular distribution of cyclin D1 protein, by retrospectively analysing cyclin D1 immunostaining patterns by IHC (anti-cyclin D1 Ab, EP12, Dako) of specimen from a cohort of 42 cyclin D1^+^ MCL cases followed at a single institution and reported in a previous study^36^. The clinical and histological features of cases were compared according to their cyclin D1 staining patterns (nuclear *vs*. nuclear + cytoplasmic) were compared (Table 1).

### Cells culture and transfection

The MCL cell lines have been described elsewhere^37^. CM cells, used as a control, are immortalised and untransformed mature B cells. Cell lines were maintained in culture in RPMI 1640 medium (Lonza) supplemented with 10–20% foetal calf serum (PAA Laboratories), 2 mM L-glutamine and antibiotics (Lonza), under a humid atmosphere at 37 °C. Cell authentication was based on short tandem repeat (STR) profiling (IdentiCell, Aarhus, Denmark).

The pEGFP-N1 plasmid was purchased from Clontech Laboratories Inc., and the pD1a-EGFP and pD1b-EGFP^38^ plasmids were kindly provided by D.A. Solomon (USCF, San Francisco, CA). Both were sequenced to check the integrity of the coding sequence. Plasmids were amplified and purified (QIAGEN Plasmid Midi Kit). JVM2 cells (7 × 10^6^ cells) were cultured without antibiotics for 24 h. Cells were resuspended in 100 μl of Nucleofector Solution V (Lonza) containing 2 μg of purified plasmids. JVM2 cells were transfected with a Nucleofector II device (Lonza) using the A-023 program. After transfection, cells were transferred to culture plates in complete culture medium and assayed for migration 48 h later as described below. At that time, the transfection efficiency (recorded by the number of GFP^+^ cells) was verified using the NucleoCounter TM-3000 according to the recommendations of the supplier (Chemometec).

### Transwell migration and invasion assays

Cultured MCL cells (5 × 10^5^ cells per insert) were suspended in RPMI 1640 medium containing 0.5% bovine serum albumin (BSA) and used to seed the top chamber of Transwell inserts (Millicell Hanging Cell Culture Inserts 5 μm PET, Millipore). The inserts were transferred to wells containing RPMI 1640 medium supplemented with recombinant SDF1 (200 ng/ml, R&D Systems) as a chemoattractant, as previously described^23^. As a control for assay specificity, SDF1-free medium was added to the lower chamber. Plates were then incubated for 4 h at 37 °C, and cells migrating to the lower chambers were counted by flow cytometry. The accuracy of cell counting was checked by adding fluorescent particles to the chambers (Accucount fluorescent particles, SpheroTech).

For invasion assays, 5 × 10^5^ cells in serum-free medium plus 0.5% BSA were added to the top chamber of 24-well inserts (Costar Transwell Permeable Support, pore size 5 μm) coated with ECM obtained from Engelbrecht-Holm-Swarm mouse sarcoma (Sigma-Aldrich). Medium supplemented with SDF1 (200 ng/ml) or without supplementation (for control), was added to the bottom chamber. Plates were incubated for 24 h at 37 °C. The invading cells present in the lower chambers were counted by flow cytometry.

LMB (L2913) was purchased from Sigma-Aldrich, and KPT-330 (S7232) from SelleckChem. For migration and invasion assays, cells were treated with either 5 ng/ml LMB or 300 nM KPT-330 for 4 h before their addition to the Transwell inserts.

### Indirect immunofluorescence, F-actin staining and confocal microscopy analysis

MCL cells were cytospun, fixed in 4% paraformaldehyde and permeabilized in 0.5% Triton-X100. The slides were then stained with an anti-cyclin D1 Ab (sc-718, Santa Cruz Biotechnology), and then with Alexa Fluor 633-conjugated goat anti-rabbit IgG (in green) and counterstained with DAPI (4′,6-diamidino-2-phenylindole dihydrochloride, Molecular Probes, in blue). Slides were observed with a confocal fluorescence microscope (Fluoview FV 100, Olympus). For XPO1 inhibition, cells were treated with KPT-330 for 4 h before IF analysis.

Cultured cells were either left untreated or treated with 200 ng/ml SDF1 to stimulate chemotaxis. Fixed and permeabilized cells were incubated with rhodamine-phalloidin, as recommended by the supplier (Molecular Probes) and analysed by fluorescence microscopy. In some experiments, SDF1-treated cells were also treated with 300 nM KPT-330 (or vehicle) for 4 h before F-actin staining.

### Immunoprecipitation and immunoblotting

Whole-cell protein extracts were prepared from cultured cells with the M-PER Mammalian Protein Extraction Reagent (Pierce Biotechnology) according to the manufacturer’s instructions. Fractions enriched in cytosol were prepared with a lysis buffer containing 1% NP40, 20 mM Tris pH 7.6, 120 mM NaCl, 1 mM EDTA and a cocktail of protease inhibitors (cOmplete ULTRA tablets, Roche). Cytoplasmic and nuclear extracts were prepared with the Nuclear/Cytosol Fractionation Kit (BioVision). Extract purity was checked by IB with Abs directed against the strictly nuclear PARP protein, and the endoplasmic reticulum-bound BiP protein. The methods used for IB and IP have been described in details elsewhere^37^.

### Affinity purification, mass spectrometry, protein characterization

These methods are described in details in the Supplementary Information.

### Engraftment of GFP/mCherry-Luc expressing MCL cells in immunocompromised mice

*In vivo* experiments were conducted in accordance with the recommendations of the EEC (86/609/CEE). They were approved by the Ethics Committee for Animal Experimentation of our institutions (*Comité Normandie d’éthique en matière d’expérimentation animale* (CENOMEXA) agreement N109-11-12/32/11-15 in France and *Hospital Clínic Ethics Committee* (IRB, reg.num. 2012/7498) in Spain). Z138-GFP-Luc, REC1-GFP-Luc, JeKo1-mCherry-Luc cell lines were generated by infection with lentivirus or retrovirus vectors (see Supplementary Information). Male SCID (n = 5–7 per group) and NSG (n = 6 per group) mice (age 6–8 weeks) received an i.v. injection of 10^7^ cells in a final volume of 100 μl. Tumour engraftment was assessed weekly by BLI. Mice received an i.p. injection of 75 mg/kg of D-luciferin and were then examined with a PhotonIMAGER (Biospace lab) for 5 min. Ventral plus dorsal emissions were captured through the whole mouse body. When the mice were killed, their infiltrated organs were collected. Single-cell suspensions were prepared and stained with IOTest CD20-PE (phycoerythrin, IM1451, FL2) or anti-CD45-VioBlue (clone 5B1, Miltenyi Biotech., FL9) Abs, or with the corresponding isotype controls. Cells were also directly analysed for GFP or mCherry fluorescence by flow cytometry. In addition, BM biopsy specimens were fixed in 4% formalin and embedded in paraffin for IHC analyses according to previously published procedures^37^. Human tumour B-cell detection procedures were performed on consecutive tissue sections, with anti-cyclin D1 (EP12) and anti-CD20 (L26) primary Abs (Dako). Preparations were examined under an Olympus DP70 microscope equipped with of a 40/0.75 NA objective and DPManager software v2.1.1 (Olympus).

For analysis of the effects of KPT-330 on cell engraftment, SCID mice (n = 10) received i.v. injections of JeKo1-mCherry-Luc cells as described above. On the day of the injection, the mice were assigned to two groups receiving either orally a 10 mg/kg dose KPT-330 or i.p. an equal volume of vehicle twice weekly. Mice underwent BLI once per week, beginning one week after cell injection. When the mice were killed, their infiltrated organs were removed and processed for flow cytometry and IHC analyses.

### Statistical analyses

The Student’s *t-*test was used to determine the significance of differences between two experimental groups. Data were analysed in two-tailed tests, with *p* < 0.05 (*) considered to be significant. Fisher’s exact test was used to evaluate the distribution of the various histological and clinical parameters between MCL cases as a function of cyclin D1 immunostaining pattern.

## Electronic supplementary material

Supplementary Information

Table S1

Table S2

Table S4
