# Supplementary material for: Cytoplasmic cyclin D1 controls the migration and invasiveness of mantle lymphoma cells
Source: Sci Rep. 2017 Oct 24;7:13946. doi: 10.1038/s41598-017-14222-1 (PMC5654982; doi:10.1038/s41598-017-14222-1)
Supplement: Supplementary file 1 — Supplementary Information [file 41598_2017_14222_MOESM1_ESM.pdf]

Supplementary information

Cytoplasmic cyclin D1 controls the migration and invasiveness of mantle lymphoma cells

Simon Body[1], Anna Esteve-Arenys[2], Hadjer Miloudi[1], Clara Recasens-Zorzo[2], Guergana Tchakarska[1,¶], Alexandra Moros[2], Sophie Bustany[1], Anna Vidal-Crespo[2], Vanina Rodriguez[2], Régis Lavigne[3], Emmanuelle Com[3], Isolda Casanova[4,5], Ramón Mangues[4,5], Oliver Weigert[6], Alejandra Sanjuan-Pla[7], Pablo Menéndez[5,8], Bénédicte Marcq[9], Jean-Michel Picquenot[1,9], Patricia Pérez-Galán[2], Fabrice Jardin[1,10], Gaël Roué[2] and Brigitte Sola[1,*]

Supplementary Methods

Antibodies

Abs against cyclin D1 (sc-718), β-actin (sc-4778), β-tubulin (sc-9104), and XPO1 (sc-5595) were purchased from Santa Cruz Biotechnologys. An Ab against α-tubulin (T6199) was purchased from Sigma-Aldrich. ImmunoPure goat anti-rabbit and anti-mouse IgG (H+L) peroxidase-conjugated Abs were purchased from Pierce Protein Research Products.

Transduction of fusion TAT-cyclin D1 proteins and analysis of transduced cells by transmission electron microscopy

The procedures for plasmids construction, fusion proteins production, purification and transduction have been described in details elsewhere, together with the electron microscopy procedures[1].

Adhesion assay

Cell adhesion on fibronectin or HS-5 stromal cells was assessed with the Vybrant™ Cell Adhesion Assay Kit (V-13181, Molecular Probes) as previously described[2].

Construction of GFP/mCherry and luciferase-expressing MCL cell lines

Phoenix™ Ampho cells and 293T cells were maintained in DMEM supplemented with 10% FBS at 37°C, under an atmosphere containing 5% CO_2 . Retroviral particles were generated by plating 3 × 10^6 Phoenix™ cells per 10 cm dish. On the day after plating, cells were transfected with 10 µg of pMSCV-Luc2-PKG-Neo-IRES-GFP plasmid[3], by the calcium phosphate method (CalPhos Mammalian Transfection Kit, Clontech), according to the kit manufacturer's protocol. The medium was changed 12 h after transfection. The viral supernatant was collected 48 h post-transfection, passed through a filter with 0.45 µm pores and used imediately. Z138 (10^6) cells were plated in 24-well plates and incubated with retroviral particles and polybrene (4 µg/ml). Cells were centrifuged at 2,200 rpm for 2 h at 32°C. Transduced cells were sorted based on the basis of their GFP expression, in a FACSAria cell sorter.

For the generation of lentiviral particles, 3 × 10^6 293T cells were plated per 10 cm dish. On the day after plating, cells were transfected with 0.75 µg of VSV-G, 1.5 µg of p8.91 and 2.25 µg of pSIN-DUAL-Luc-GFP2 (ref. 4), with the Lipofectamine™ 2000 (Invitrogen) system, according to manufacturer's protocol. The medium was changed 12 h post-transfection. The viral supernatant was collected 48 h post-transfection, passed through a filter with 0.45 µm pores (Millex®, Millipore) and used immediately. REC1 cells (2 x 10^5) were plated in 24-well plates and incubated with lentiviral particles. Transduced

cells were sorted on the basis of their GFP expression in a FACSAria cell sorter. Luciferase activity was measured with the Luciferase Assay System (Promega).

JeKo1 cells were transfected with lentiviruses carrying the luciferase gene. Briefly, we cotransfected 293T cells with FUW-Luc-mCherry-puro, VSV-G and psPAX2, using Lipofectamine™ 2000. The supernatant was harvested 24 or 48 h later, passed through a filter with 0.45 µm pores and 50 µl of the filtrate was used for the spininfection of 5 x 10^6 JeKo1 cells in the presence of 8 µg/ml polybrene (Sigma) and 10 nM HEPES (Gibco), as previously described[5]. Stably transduced cell lines were selected in medium containing 1 µg/ml puromycin (Sigma-Aldrich)

Immunoprecipitation of cyclin D1-interacting proteins and separation by electrophoresis

We obtained cell extracts enriched in cytosolic proteins by lysing cultured JeKo1 and U266 cells (5 x 10^7 cells) in an IP buffer containing 50 mM Tris pH 7.5, 150 mM NaCl, 1% NP40, 1 mM sodium orthovanadate and a cocktail of protease inhibitors (P-8340, Sigma-Aldrich). We then subjected these extracts (5 mg of protein) to immunoprecipitation with 10 µg of anti-cyclin D1 Ab (sc-718, Santa Cruz Biotechnology). This Ab is directed against the canonical form of cyclin D1 expressed in MCL and some MM cells. Samples were diluted in detergent-free Laemmli buffer and proteins were separated by SDS-PAGE in a 12% precast gel (Gebagel; Gene Bio-Applications, Paris, France). The gel was fixed by incubation in 30% ethanol, 10% acetic acid for 15 min, washed in MilliQ water for 15 min and stained with Coomassie blue, with the EZBlue gel-staining reagent (Sigma-Aldrich).

Trypsin digestion of proteins

Gel lanes were manually cut into 20 pieces, which were then processed by two successive rounds of washing in 50 mM NH_4HCO_3 in ACN (acetonitrile)/H_2O (v/v), rehydration in 100 mM NH_4HCO_3, and dehydration in 100% ACN. Gel slices were subjected to reduction in 65 mM DTT (dithiothreitol) at 37°C, followed by alkylation in 135 mM iodoacetamide in the dark at room temperature. Gel slices were then washed/dehydrated/rehydrated as before and subjected to trypsin digestion (modified trypsin from Promega France, 4 ng/ml in 50 mM NH_4HCO_3) overnight at 37°C. The digested peptides were then extracted in two successive steps involving the addition of ACN/H_2O/trifluoroacetic acid (TFA) (70/30/1). Supernatants were collected and concentrated in a SpeedVac.

Mass spectrometry analysis

Mass spectrometry (MS) was performed with a nanoflow high-performance liquid chromatography (HPLC) system (LC Packings Ultimate 3000, Dionex) connected to a hybrid LTQ-OrbiTrap XL (Thermo Fisher Scientific) equipped with a nanoelectrospray ion source (New Objective, Woburn). Mobile phases A (99.9 % MilliQ water and 0.1% formic acid (v:v)) and B (99.9% acetonitrile and 0.1% formic acid (v:v)) for HPLC were delivered by the Ultimate 3000 nanoflow LC system (LC Packings, Dionex). We loaded 10 µl of prepared peptide mixture onto a trapping precolumn (5 mm × 300 µm i.d., 300 Å pore size, Pepmap C18, 5 µm), through which 2% buffer B was passed for three minutes, at a flow rate of 25 µl/min. This step was followed by reverse-phase separations at a flow rate of 0.250 µl/minute on an analytical column (15 cm × 300 µm i.d., 300 Å pore size, Pepmap C18, 5 µm, Dionex). A gradient of 2% to 90% buffer B was passed through the column for 105 min. The column was then washed with 90% buffer B for 16 min, and with 2% buffer B for 19 min

before the loading of the next sample. Peptides were detected by direct elution from the HPLC column into the electrospray ion source of the mass spectrometer. An ESI voltage of 1.5 kV was applied to the HPLC buffer *via* the liquid junction provided by the nanoelectrospray ion source, and the ion transfer tube temperature was set at 200°C.

The MS instrument was operated in its data-dependent mode, with automatic switching between full-scan survey MS and consecutive MS/MS acquisition. Full-scan survey MS spectra (mass range 400 – 2000) were acquired in the OrbiTrap section of the instrument, with a resolution of R = 60,000 at m/z 400; ion injection times were calculated for each spectrum so as to allow the accumulation of 10^6 ions in the OrbiTrap. The seven peptide ions yielding the most intense signals in each survey scan, with an intensity above 2,000 counts (to avoid triggering fragmentation too early during the peptide elution profile) and a charge state ≥ 2, were sequentially isolated at a target value of 10,000 and fragmented in the linear ion trap by collision-induced dissociation (CID). Normalised collision energy was set to 35%, with an activation time of 30 ms. Peaks selected for fragmentation were automatically placed on a dynamic exclusion list for 120 s, with a mass tolerance of ± 10 ppm to avoid prevent selection of the same ion for fragmentation more than once. The following parameters were used: the repeat count was set to 1, the exclusion list size limit was 500, singly charged precursors were rejected and the maximum injection time was set at 500 ms and 300 ms for full MS and MS/MS scan events, respectively. For an optimal duty cycle, the fragment ion spectra were recorded on the LTQ mass spectrometer in parallel with OrbiTrap full-scan detection. For OrbiTrap measurements, external calibration was performed before each injection series, to ensure an overall mass accuracy error of less than 5 ppm for the detected peptides. MS data were saved in a RAW file format, with XCalibur 2.0.7 and tune 2.4 (Thermo Fisher Scientific).

Data processing and identification of peptides and proteins

Data were analysed with Proteome Discoverer 1.2 software supported by Mascot (Matrixscience) and SEQUEST database search engines for peptide and protein identification. MS/MS spectra were compared with the SwissProt Database filtered with *Homo sapiens* taxonomy (SwissProt release 57; 428,650 sequences, 154,416,236 residues), for protein identification with Proteome Discoverer software (version 1.2.0.208). Mass tolerance was set to 10 ppm for MS and 0.5 Da for MS/MS. Trypsin selectivity was set to full, with one miscleavage allowed. The fixed and variable modifications allowed were the carbamidomethylation of cysteines and oxidation of methionine, respectively. The peptides identified were filtered on the basis of their Xcorr values (Sequest algorithm) and Mascot scores, to obtain a false discovery rate (FDR) of 1% at the peptide level, with the Mascot decoy search strategy, corresponding to a mean false positive rate of 5%. Protein coverage, corresponding to the percentage of the protein's sequence represented by the peptides identified, was determined. The validated Xcorr and Mascot search results for the two MS analyses of all the elution fractions were merged and two protein lists corresponding to each cell line were created (Table S1 and S2).

Datamining

For the validation of mass spectrometry results, the two protein lists generated were compared with each other and with the list of proteins interacting with cyclin D1 in the Granta MCL cell line[6]. The proteins present in the three cell lines were then selected and analysed for gene ontology (GO) clustering, with DAVID Bioinformatics Resources[7]. For analysis of the cyclin D1 interactome in JeKo1 cells, we selected the 200 proteins with the

highest peptide coverage percentages (Table S4) for further analysis with DAVID tools and then with the PANTHER database[8], to determine their cellular function, associated biological process, cell component, and protein class. Possible protein-protein interactions were sought by using the selected 51 cytoskeleton-associated proteins to query the STRING database[9]. The data are summarized as a network showing current interactions, with network nodes representing proteins, and edges representing protein/protein interactions.

Supplementary figures

Supplementary Fig. S1

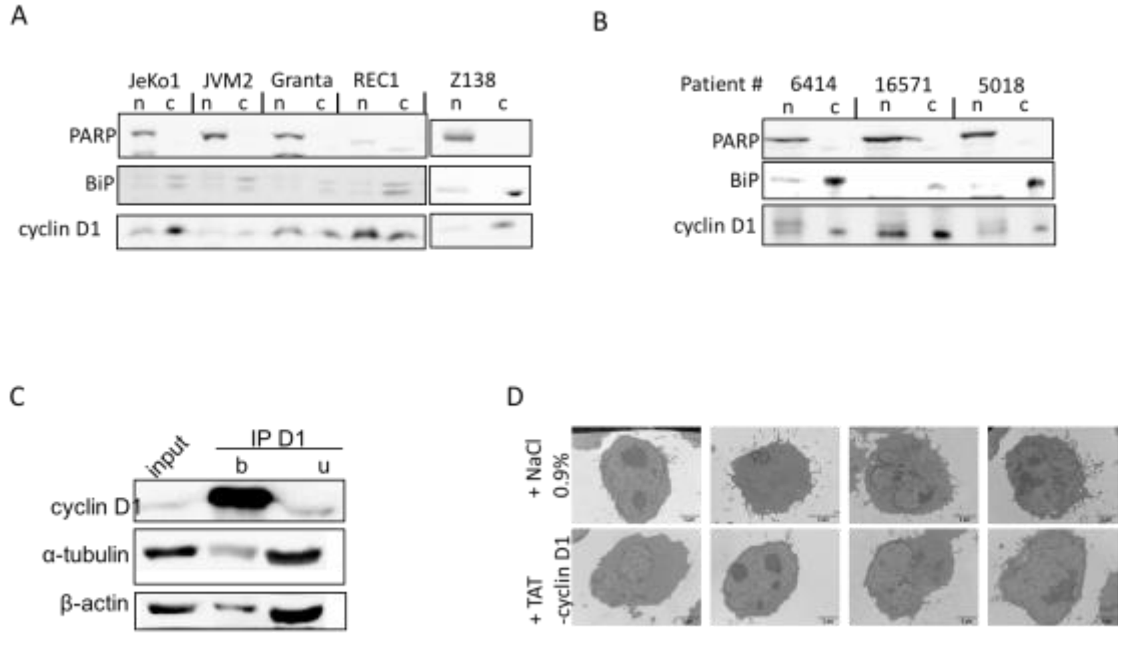

A, cultured MCL cells were harvested. Nuclear (n) and cytosolic (c) protein extracts were prepared with the BioVision kit. The purified proteins were separated by SDS-PAGE and analysed by IB with the Abs indicated. The purity of each fraction was checked with Abs against PARP and BiP with these proteins also used as controls for gel loading. **B**, tumour cells from the indicated patients were purified. Nuclear and cytoplasmic fractions were obtained, separated by SDS-PAGE and the proteins were transferred onto nitrocellulose membranes. The membranes were incubated with the indicated Abs, which were also used to determine extract purity. **C**, proteins purified from JeKo1 cells were immunoprecipitated with an anti-cyclin D1 Ab. The bound (b) and unbound (u) fractions were resolved by SDS-PAGE and analysed by IB with anti-cyclin D1, anti-α-tubulin, and anti-β-actin Abs. An aliquot of the purified protein preparation (1/10) was analysed directly (input). **D**, RAMOS cells were transduced with 250 ng/ml TAT-cyclin D1 protein for 6 h (or treated with 0.9% NaCl as a control) and analysed by transmission electronic microscopy, as previously described[1]. The corresponding scale bars are indicated on each image.

Supplementary Fig. S2

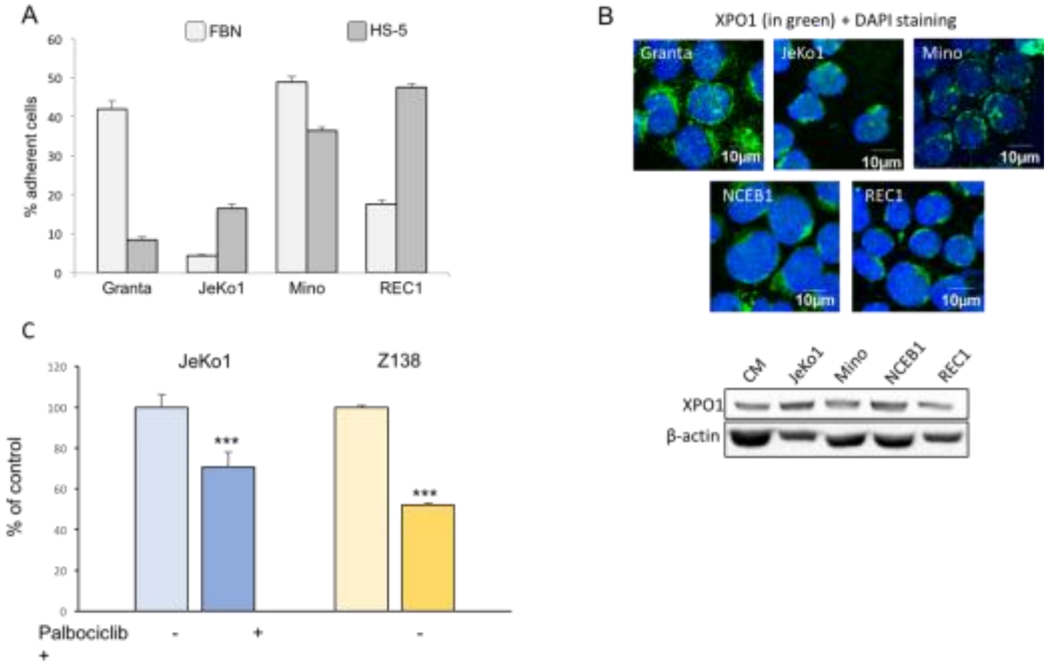

Fig. S2

A, we coated 96-well plates with fibronectin (FBN) or used them as culture plates for HS-5 stromal cells. MCL cells were stained with calcein-AM and allowed to adhere to substrates for 4 h. Fluorescence was recorded before and after thorough washing, and the percentage of adherent cells was calculated and plotted on the graph (means ± s.d. for three independent experiments carried out in triplicate). **B**, cultured MCL cells were cytospun, fixed and permeabilized. They were then stained with an anti-XPO1 (sc-5595, Santa Cruz Biotechnologies) primary Ab and an AlexaFluor 488-conjugated goat anti-rabbit IgG (Life Technologies) secondary Ab. Nuclei were counterstained with DAPI. Cells were analysed by confocal microscopy (Fluoview FV 1000 confocal microscope and Fluoview Viewer software, Olympus) at x 180 magnification. Whole-cell extracts were obtained and the proteins they contained were separated by electrophoresis and analysed by IB with the same anti-XPO1 Ab and an anti-β-actin Ab as a control. **C**, JeKo1 and Z138 cells were treated with 2 µM palbociclib (+) or vehicle (-) for 24 h and assayed for chemotaxis. Triplicate samples from two independent experiments were analysed. The percentages of migrating cells in palbociclib-treated samples and vehicle-treated samples were calculated. The means ± s.d. are plotted on the graph. ***, $p < 0.001$.

Supplementary Tables

Table S1. List of the proteins identified as interacting with cyclin D1 in JeKo1 cells (Table S1_R1.xls)

Table S2. List of the proteins identified as interacting with cyclin D1 in U266 cells (Table S2_R1.xls)

Table S4. List of the top 200 most represented proteins interacting with cyclin D1 in JeKo cells (Table S4_R1.xls)

Table S3. Cyclin D1-interacting proteins in JeKo1 cells present in Granta and U266 cell lines

Cell cycle	Protein folding	DNA repair	DNA replication	Transcription regulation	Cell structure	Development	Metabolism	RNA metabolism	Miscellaneous
CDK1	AHSA1	MSH6	MYBBP1A	BBX	CAMSAP3	ATXN10	ALDOA	ABCE1	CSE1L
CDK2	BAG2	PCNA	RFC1	GTF3C1	CFL1		ATP5B	DDX24	COG7
CDK3	CCT2	RAD51	RFC2	GTF3C4	DYNC1H1		CAD	DDX52	DBC1
CDK4	CCT3		RFC4	HDAC1	ELMO1		ENOA	EEF1A1	DNMT1
CDK5	CCT4		RFC5	HDAC2	KRT8		FASN	GNC1L1	GPATCH8
CDK5RAP2	CCT5			SFRS14	LMNB2		HADHA	NONO	PPP2R1A
CDK6	CCCT6A			ZFP106	VIM		MDH2	PCBP1	RNF2
CDKN1A	CCT7						OGT	PCBP2	RNF219
CDKN1B	CCT8						PRDX6	SNRNP40	TGM3
CDKN1C	DNAJA1								VDAC1
CDKN2C	DNAJA2								
MKI67	DNAJB6								
RB1	HSP9								
WDR5	HSP90AA1								
	HSPA1L								
	HSPD1								
	HSPH1								
	PPIA								
14 (17%)	18 (22%)	3 (4%)	5 (6%)	7 (8%)	7 (8%)	1 (1%)	9 (11%)	9 (11%)	10 (12%)

Whole cell proteins were prepared from cultured JeKo-1 and U266 cell lines and incubated with anti-cyclin D1 antibody. Cyclin D1-associated complexes were run into gels, separated and analyzed by mass spectrometry. Cyclin D1-interacting proteins common to both cell lines and present in Granta cells, according to Jirawatnotai *et al.* (2011), were recorded and classified with DAVID tools (Huang et al., 2009). Strictly identical genes are noted in black, genes belonging to the same family in grey. Well-known cyclin D1 interactors such as CDK4, CDK6, CDK2, PCNA, RB1, p27 (CDKN1B) were characterized as well as novel proteins.

Table S5. Organs infiltration at the end of *in vivo* experiments

Mouse strain	Cell line	Spleen	Bone marrow	Blood	Brain	# of mice with a least one luciferase activity focus
SCID	JeKo1-mCherry-Luc	5/6	2/6	0/6	4/6	6/7*
	Z138-GFP-Luc	4/5	4/5	0/5	5/5	5/5
	REC1-GFP-Luc	4/5	3/5	1/5	1/4	6/6
NSG	JeKo1-mCherry-Luc	3/3	3/3	1/3	6/6	6/6
	Z138-GFP-Luc	3/3	3/3	0/3	5/5	6/6
	REC1-GFP-Luc	1/3	2/3	0/3	2/6	6/6

MCL cell lines were engineered to express the luciferase gene (Luc) and/or a fluorescent protein GFP or mCherry. MCL cells were injected in the caudal vein of either SCID or NSG mice. The overall distribution of MCL tumor cells was determined by BLI during the time course of the experiment and confirmed by flow cytometry from isolates of hematopoietic and non-hematopoietic organs at the time of mice euthanasia. *In this series one mouse died for an unknown reason before the onset of the disease.

Supplementary References

1. Tchakarska, G., Roussel, M., Troussard, X. & Sola, B .Cyclin D1 inhibits mitochondrial activity in B cells. *Cancer Res*. **71**, 1690-1699 (2011).

2. Bustany, S. *et al*. Cyclin D1 unbalances the redox status controlling cell adhesion, migration, and drug resistance in myeloma cells. *Oncotarget*. **7**, 45214-45224 (2016).

3. Zuber, J. *et al*. Mouse models of human AML accurately predict chemotherapy response. *Genes Dev*. **23,** 877–889 (2009).

4. Bosch, R. *et al*. A novel inhibitor of focal adhesion signaling induces caspase-independent cell death in diffuse large B-cell lymphoma. *Blood*. **118**, 4411-4420 (2011).

5. Jacobson, C. *et al*. HSP90 inhibition overcomes ibrutinib resistance in mantle cell lymphoma. *Blood*. **128**, 2517–2526 (2016).

6. Jirawatnotai, S. *et al*. A function for cyclin D1 in DNA repair uncovered by protein interactome analyses in human cancers. *Nature*. **474**, 230-234 (2011).

7. Huang, D.W., Sherman, B.T. & Lempicki, R.A. Systematic and integrative analysis of large gene lists using DAVID bioinformatics resources. *Nat. Protoc*. **4**, 44-57 (2009).

8. Mi, H., Muruganujan, A. , Casagrande, J.T. & Thomas, P.D. Large-scale gene function analysis with the PANTHER classification system. *Nat. Protoc*. **8**, 1551-1566 (2013).

9. Szklarczyk, D. *et al*. The STRING database in 2017: quality-controlled protein-protein association networks, made broadly accessible. *Nucl. Acids Res*. **45**, D362-D368 (2017).
